# Anticancer therapeutic potential of genus *Diospyros*: From phytochemistry to clinical applications—A review

**DOI:** 10.1002/fsn3.4375

**Published:** 2024-08-13

**Authors:** Abdur Rauf, Zuneera Akram, Nabia Hafeez, Anees Ahmed Khalil, Ahood Khalid, Zoya Abid, Hassan A. Hemeg, Abdullah S. M. Aljohani, Waleed Al Abdulmonem, Mohammed Mansour Quradha

**Affiliations:** ^1^ Department of Chemistry University of Swabi Anbar Khyber Pakhtunkhwa Pakistan; ^2^ Department of Pharmacology, Faculty of Pharmaceutical Sciences Baqai Medical University Karachi Pakistan; ^3^ Center of Biotechnology and Microbiology University of Peshawar Peshawar Pakistan; ^4^ University Institute of Diet and Nutritional Sciences, Faculty of Allied Health Sciences The University of Lahore Lahore Pakistan; ^5^ Department of Clinical Laboratory Sciences, College of Applied Medical Sciences Al‐Madinah Al‐Monawara Saudi Arabia; ^6^ Department of Medical Biosciences, College of Veterinary Medicine Qassim University Buraydah Saudi Arabia; ^7^ Department of Pathology, College of Medicine Qassim University Buraydah Saudi Arabia; ^8^ College of Education Seiyun University Seiyun Hadhramawt Yemen

**Keywords:** angiogenesis, anticancer agents, apoptosis, cellular proliferation, Diospyros, ethnopharmacology, in silico studies, in vitro studies, in vivo studies

## Abstract

The genus *Diospyros* has gained significant attention in the scientific community owing to its diverse bioactivities ascribed to specific bioactive constituents present in different species of this plant. Phytochemicals like flavonoids, terpenoids, and xanthones have been reported to be present in other *Diospyros* species responsible for their pharmacological properties. These compounds are well known for their diverse potent therapeutic potentials, such as antimicrobial, antioxidant, anti‐inflammatory, and anticancer properties. This review enlightens the details of the Genus Diospyros, ranging from an overview of its species to an in‐depth analysis of phytochemistry, ethnopharmacology, and their potential as anticancer agents. Different species, including *Diospyros lotus*, *Diospyros kaki*, *Diospyros maritima*, *Diospyros mespiliformis*, and *Diospyros tricolor*, presented with an enormous range of anticancer activities against human cancer cell cultures. Moreover, this review highlights the results of various in vitro (antiproliferative, cytotoxic effects against), in vivo (inhibition of tumor, apoptosis), and *in silico* (GLU234, GLU278, and LYS158 protein residues) studies, elucidating its preclinical anticancer potential. The anticancer potential displays inhibition of cellular proliferation, induction of apoptosis, and mitigation of angiogenesis. Furthermore, this review may elaborate the use of traditional knowledge, modern research, and potential therapeutic applications in the field of anticancer ethnopharmacology. As the modern‐day research approaches novel alternatives to combat diseases like cancer, the Genus Diospyros may emerge as a promising avenue with the potential to yield innovative and effective therapeutic agents.

## Graphical abstract

The Genus Diospyros has garnered significant scientific interest due to its diverse bioactivities, attributed to various phytochemicals like flavonoids, terpenoids, and xanthones. Several species, including *D. lotus*, *D. kaki*, and *D. tricolor*, exhibit promising anti‐cancer properties through in vitro and in vivo studies, demonstrating inhibition of proliferation, induction of apoptosis, and mitigation of angiogenesis. Integrating traditional knowledge with modern research, Diospyros shows potential as a source for novel and effective anti‐cancer therapeutics in ethno‐pharmacology.

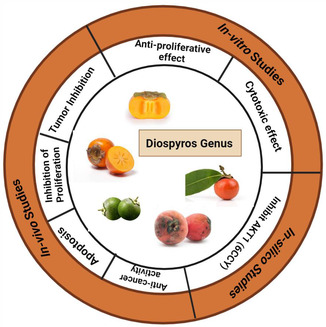

## 1 INTRODUCTION

Cancer is a complicated disease that results in uncontrolled tumor cell proliferation due to signaling failure of oncogenic expressions. Since tumors can begin in any organ, the resulting malignancies are highly diverse (Anusewicz et al., 2020). In 2020, cancer was anticipated to result in over 10 million fatalities and 19.3 million new diagnoses (Sung et al., 2021). Breast cancer accounts for approximately 11.7% of all new cancer diagnoses in women annually (Bray et al., 2018). Until now, efforts have been made to create effective methods for diagnosing and treating cancer. Among the available therapeutic methods are chemotherapy (Dickens & Ahmed, 2018), molecularly targeted therapy (Piawah & Venook, 2019), gene therapy (Carrillo et al., 2018), radiation (Dobosz & Dzieciątkowski, 2019), immunotherapy (Chang et al., 2019), and phototherapy (Marabini et al., 2020). Indirectly, medicinal plant therapeutic compounds also help improve overall health by enhancing cellular signaling processes and activating endogenous defense mechanisms (Gopal et al., 2011). There are 240 species in the ubiquitous tropical genus *Diospyros*. Of those species, 59 are found in India, Japan, Thailand, South Africa, the Philippines, and Nigeria (Alex et al., 2012). The medium‐sized Gürke, the Gaub persimmon, is an endemic perennial to India. An ethnomedicinal plant called *Diospyros peregrina* (Gaertn.) Gürke's alcoholic fruit extract has hypoglycemic, diuretic, and anticancer properties (Philander, 2011). Several parts of the plant have a variety of medicinal uses outside its traditional usage for dysentery and menstrual problems. The fruit of *D. peregrina* contains soluble tannins, peregrinol, flavonoids, hexacosanol, hexacosane, betulinic acid, β‐sitosterol, and lupeol. *D. peregrina* was previously discovered to have anticancer properties by experimenting on mice with Ehrlich ascites carcinoma (Alex et al., 2012; Philander, 2011).

Herbalists in South Africa's Western Cape, which has a lot of different plants, said they used *Dioscorea villosa* root to treat diarrhea, intestinal worms, and too much flatulence (De Wet & Ngubane, 2014). Moreover, the root of the *D. villosa* plant was utilized to alleviate pain and dysmenorrhea in a rural area of northern Maputaland (Rajesh et al., 2017). Nanoparticles (NPs) isolated from the leaves of another plant, *Diospyros ferrea* (Wild.), also exhibited anticancer activity against MCF‐7 (Michigan Cancer Foundation‐7) cancer cell lines (Park et al., 2017). Investigations into the pharmacology of the calyx of *Diospyros kaki* Thunb. (DKC) have not been entirely explained, despite reports that it has significant polyphenol levels. DKC's anticancer efficacy and putative molecular mechanism against human colorectal cancer cells were analyzed using the MTT (3‐(4,5‐dimethylthiazol‐2‐yl)‐2,5‐diphenyltetrazolium bromide) test. Extracts of the tree *Diospyros kaki* calyx were tested for their ability to inhibit cell proliferation in 70% ethanol (DKC‐E70). Western blotting and reverse transcription polymerase chain reaction (RT‐PCR) were used to examine the effect of DKC‐E70 on cyclin D1 messenger RNA (mRNA) and protein expression. Results revealed that DKC‐E70 inhibited LoVo (epithelial cell line), SW480 (human colon adenocarcinoma cell line), and HT‐29 (human colorectal adenocarcinoma cell line). Although DKC‐E70 decreased cyclin D1 expression at both the protein and mRNA levels, the downregulation of cyclin D1 protein by DKC‐E70 may have been caused by the induction of cyclin D's degradation and transcriptional inhibition.

Furthermore, it was observed that the effect of DKC‐E70 on cyclin D1 degradation was diminished in the presence of MG132 (proteasome inhibitor). Moreover, DKC‐E70 phosphorylated cyclin D1's threonine‐286 (T286) and T286 mutated to alanine (T286A) reversed DKC‐downregulation E70's of cyclin D1. The phosphorylation of T286 by DKC‐E70 and consequent degradation of cyclin D1 were also seen to be inhibited when ERK1/2 (extracellular signal‐related kinases 1 and 2), p38, or GSK3 (glycogen synthase kinase 3) inhibitors were present. DKC‐E70 suppressed the expression of β‐catenin and TCF4 (transcription factor 4) and β‐catenin/TCF‐dependent luciferase activity in cyclin D1 transcriptional inhibition. Thus, this study implies cyclin D1 as one of the potential anticancer targets that can be downregulated via DKC‐E70 through cyclin D1 breakdown. Furthermore, downregulation can be carried out by T286 phosphorylation dependent on ERK1/2, p38, or GSK3 and cyclin D1 transcriptional suppression via Wnt signaling. Hence, based on these findings, DKC‐E70 may be possible for developing chemoprevention or therapeutic medicines for human colorectal cancer.

## 2 GENUS DIOSPYROS: AN OVERVIEW

The broad pantropical genus *Diospyros* comprises several hundred species, mostly evergreen trees and shrubs. Though a few species are deciduous, inhabiting the temperate regions, its best‐known features are the genus’ delicious fruits and dark wood. It derives its name from the Greek term “Diospyros,” which means “divine wheat” or “fruit of the gods.” Despite their fibrous texture, the edible fruits of the *Diospyros* species are rich in vitamins and minerals. Increased tannin concentration makes them extremely astringent before they are fully mature. *Diospyros lotus*, a plant native to south‐eastern Europe and south‐west Asia, produces little, yellow, or purplish‐black fruits called date‐plum that soften up and become edible when ripe. Likewise, *D. kaki* (Oriental persimmon) is a long‐standing, widespread cultivated plant primarily found in China and Japan (Sarkhosh et al., 2024). This species has distinct male and female parts. The calyx lobes are frequently star‐shaped that become larger as the fruits develop into enormous, spherical berries. A persimmon cultivar grown in Israel's Plain of Sharon is known as the Sharon fruit and is sold in supermarkets. The south‐eastern American plant *Diospyros virginiana* produces the fruits of American persimmon. However, several portions of the African *Diospyros mespiliformis* are also traditionally utilized for medicinal and practical reasons. For instance, edible jackal berry fruits are produced by this species. It is utilized to create a glaze that is used on ceramics. Elephants, raccoons, gorillas, and deer eat jackal berry fruits in the wild, serving as crucial seed dispersers. Since ancient times, ebony wood has been traded. This slow‐growing *Diospyros ebenum* species is currently endangered, and its legal timber export is restricted, provided the source for this highly valued ebony. Ebony wood is incredibly tough, primarily black, solid, durable, and quite glossy when polished. Apart from furniture, it is historically used for the black pieces in chess sets and, along with ivory, as piano keys and fretboards on stringed instruments like guitars and violins. The primary source of ebony now comes from woodlands in Central Africa and is called *Diospyros crassiflora* (Sodhi et al., 2023).

Over the range of the genus *Diospyros*, numerous uses catering to needs of human beings have also been documented. Unripe fruits of the Central American and Mexican *Diospyros digyna* are used as a fish poison, while unripe fruits of the Asiatic *Diospyros oleifera* produce persimmon oil for waterproofing (Nafiu et al., 2013). *Diospyros abyssinica*, often referred to as kôforonto and baforonto, is a species found in Mali and other parts of southern Africa that belongs to the Ebenaceae family. Moreover, it is also found in Angola, Guinea, Eritrea, and Ethiopia. Ayurvedic, African, and Chinese traditional medicine are just a few conventional medical systems using trees. Almost every component of these plants has been employed in medicine in some capacity, including as an astringent and a treatment for troubled digestion. In Zimbabwe, root juice from *Albizia lebbeck* is mixed with the bark and leaves of *D. abyssinica* to treat snakebites. The triterpenoids, such as betulin, betulinic acid, and lupeol, are the chief ingredients isolated from *D. abyssinica*. These are all well‐known anti‐inflammatory substances. The usage of this species in traditional medicine demonstrates that it has excellent therapeutic significance. The antioxidant activity of *D. abyssinica* root bark has been investigated. It extracted dichloromethane, petroleum ether, 80% aqueous ethanol, chloroform, and water (at 50°C and 100°C). It was discovered that the root bark of *D. abyssinica* is the most abundant source of extracted chemicals, with antioxidants making up 36.7% of the weight of the plant material. The 80% ethanol and methanol extracts showed the highest level of radical scavenging action for *D. abyssinica*. Thus, this plant appears to be an incredible source of antioxidants (Rauf et al., 2017).

Although *Diospyros* plants are used for edible produce, valuable wood, and decorative purposes, many folk medicinal techniques have also used the plant parts of many different species to treat bleeding, incontinence, sleeplessness, hiccoughs, diarrhea, etc. Several species of this genus have been used to isolate phytochemical components, such as polyphenols, terpenoids, lupanes, hydrocarbons, ursanes, tannins, and lipids, as well as benzopyrones, oleananes, naphthoquinones, and taraxerones. In vitro, in vivo, and clinical studies have demonstrated the biological efficacy of these plants as antimicrobial, antioxidant, analgesic, inflammatory, antidiabetic, thermogenic, anthelmintic, and enzyme‐inhibiting agents. This genus is a rich source of pharmacologically relevant components and expedites drug discovery. The edible fruit‐yielding varieties are *D. kaki* (Oriental persimmon), *D. virginiana* (North American persimmon), *D. digyna* (black sapote), *D. lotus* (date‐plum), and *Diospyros rhombifolia* (princess persimmon). The copper, calcium, iron, potassium, sodium, magnesium, and zinc elements are all present in *D. kaki* fruit mineral profiles (Park et al., 2017). Black ebony (*D. ebenum* and *Diospyros melanoxylon*) and striped ebony are the species that produce lumber (*Diospyrus celebica* and *Diospyrus muns*). Certain species, including *D. crassiflora*, are carved into wood for ornamental purposes.

The *Diospyros* species are widely used in traditional medicine in the tropical areas. As a tonic, powder, and poultice, fruits, barks, leaves, hardwoods, and roots have been used to treat a variety of ailments, including asthma, dermatitis, hypertension, atherosclerosis, lumbago, biliousness, sleeplessness, and bleeding, among others. This substance has several common uses, including as astringent, as laxative for constipation, carminative, sedative, febrifuge, antihypertensive, vermifuge, and antidiuretic (Carrillo et al., 2018). For instance, the traditional Chinese medicine (TCM) drug NaoXinQing, a standardized extract of *D. kaki* leaves, treats neurological disorders (Dobosz & Dzieciątkowski, 2019). Similarly, *Diospyros lycioides* is among the several medicinal plants utilized in Botswana to treat HIV/AIDS (human immunodeficiency virus/acquired immunodeficiency syndrome) (Marabini et al., 2020); in Nigeria, *D. mespiliformis* roots have been used as antimalarial agents (Chang et al., 2019). These plants are mentioned as traditional remedies in pharmacopoeia and medical texts. The identification of numerous bioactive components from this genus, however, has been made possible by bioassay‐guided fractionation using a variety of chromatographic methods, including gas chromatography (GC), high‐performance liquid chromatography (HPLC), and capillary electrophoresis (CE), as well as metabolomics methods like mass spectrometry (MS) and nuclear magnetic resonance (NMR) (Alex et al., 2012; Gopal et al., 2011). Such potent therapeutics include naphthoquinones (diospyrin, plumbagin, 8‐hydroxyisodiospyrin, and ebenone) (De Wet & Ngubane, 2014; Philander, 2011), anthraquinones, terpenoids (lupane, taraxerol, ursane, oleanane, and lupeol), steroids, lignans, flavonoids (myricetin), naphthalene (diospyrol), phenolic acids (gallic acid, diospyric acid, ellagic acid, ursolic acid, betulinic acid, and maslinic acid), 7‐methyljuglone, and amyrin (Rajesh et al., 2017). Moreover, research using binding energy and protein–ligand docking has improved the screening of therapeutic candidates and provided insight into the biological mechanisms.

## 3 PHYTOCHEMISTRY OF GENUS DIOSPYROS

Secondary metabolites, anthraquinones, terpenoids, steroids, and tannins, were identified in phytochemical analyses of extracts from the roots, leaves, bark, and hardwood fractions of the *Diospyros lotus*. Due to numerous bioactive chemical components, the plant's medicinal value can be correlated. The plant's crude methanolic extract contained both polar and nonpolar phytoconstituents. The plant is used as a folk remedy due to bioactive phytoconstituents that require an additional study. In the present investigation, the crude methanolic extract of *D. lotus* roots contains all the polar and nonpolar constituents found in the roots. The roots have no record of the presence of anthraquinones, terpenoids, or steroids. According to the literature, the genus *Diospyros* shows pesticidal and biological activities (Chopra & Nayar, 1956; Ghias et al., 2011; Kirtikar & Basu, 1918; Pérez‐Valera et al., 2024). The principal components extracted from *Diospyros* species are triterpenes and related steroid derivatives. Chopra and Nayar (1956) identified the triterpenes betulinic acid, betulin, and ursolic acid isolated from a dichloromethane extract of *Diospyros leucomelas* Poir. leaves using 1H‐ and 13C‐NMR spectroscopy. Yoshihira et al. (1970) isolated four naphthoquinones (7‐Methyljugulone, isodiospyrin, mamegakinone, and 7‐methyljugulone tetramer) and three triterpenoids (betulinic acid, taraxerol, and oxallobetulin) from a chloroform extract of *D. lotus* (L.) root (Kirtikar & Basu, 1918).

Further research is required to ascertain whether the unique activity of *Diospyros* is due to the presence of anthraquinones, terpenoids, or steroids. Traditional medicine uses anthraquinones, terpenoids, and steroids in the root extract. The quantity of extract extracted from the leaves was negligible compared to the roots. Terpenoids and tannins were identified in crude leaf extracts prepared with methanol, ethyl acetate, and chloroform, whereas bark extracts contained terpenoids, steroids, and anthraquinones. Although no research has been documented, the presence of these compounds in the bark may have anti‐inflammatory benefits. In addition to anthraquinones, terpenoids, and steroids, hardwood extracts contained anthraquinones (Jeffreys et al., 1983). Based on spectral analysis, a new triterpene was isolated from the fruit of *D. peregrina* and identified as lup 20(29)‐en‐3a, 27 diol (Maridass, 2008). Maridass (2008) investigated the chemical composition of *Diospyros malabarica* Desr. fruit oil using capillary GC and GC/MS experiments. More than 35 isolated components were effectively named beta‐bisabolene (β‐bisabolene) (25.86%) and trans‐methyl isoeugenol (31.86%) (del Carmen Recio et al., 1995). Several anti‐inflammatory compounds, including ursolic acid, betulin, and betulinic acid, are extracted from *Diospyros leucomelas* (Xiu‐Zhen et al., 1989). Isodiospyrin, beta‐amyrin (β‐amyrin), olean‐12‐en‐3‐one, and Bi‐naphthoquinone (Alake, 1994) are responsible for cytotoxicity in *Diospyros morrisiana* (Alake, 1994). *D. tricolor* was used to isolate diosquinone, an antibacterial compound (Loder et al., 1957). Due to phenolic compounds, *Diospyros mollis* is an effective anthelmintic (Chen et al., 2024; Marston et al., 1984). 7‐Methyljuglone, mamegakinone, and isodiospyrin are antifungal and molluscicidal compounds found in *Diospyros usambarensis* (Bouzayani et al., 2022). For phytochemical analysis of the plant's roots, stems, bark, leaves, and unripe fruit, *D. malabarica* extracts were prepared using water, methanol, ethanol, ethyl acetate, dichloromethane, and petroleum ether. All plant extracts were analyzed qualitatively for phytochemical constituents, such as flavonoids, tannins, terpenoids, and saponins, and quantitatively for total flavonoid content (TFC) and total phenol content (TPC). Table 1 shows phytochemical and chemical structures present in different species of *Diospyros*.

**TABLE 1:** Phytochemicals and their chemical structures.

Species	Class of phytochemical	Phytochemicals	Chemical structures
*D. lotus*	Pentacyclic triterpene	1. Betulinic acid	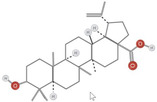
		2. Taraxerol	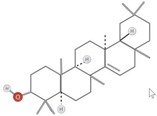
*D. leucomelas*	Pentacyclic triterpene	1. Betulinic acid	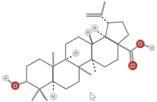
		2. Betulin	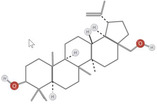
		3. Ursolic acid	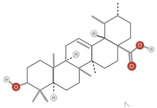
*D. peregrina*	Pentacyclic triterpene	1. Lup 20(29)‐ene‐3beta, 27 diol	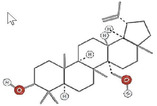
*D. malabarica*	Sesquiterpenoids	1. β‐Bisabolene	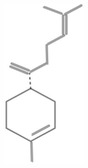
	Dimethoxybenzenes	2. Trans‐methyl isoeugenol	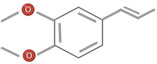
*D. morrisiana*	Naphthoquinone	1. Isodiospyrin	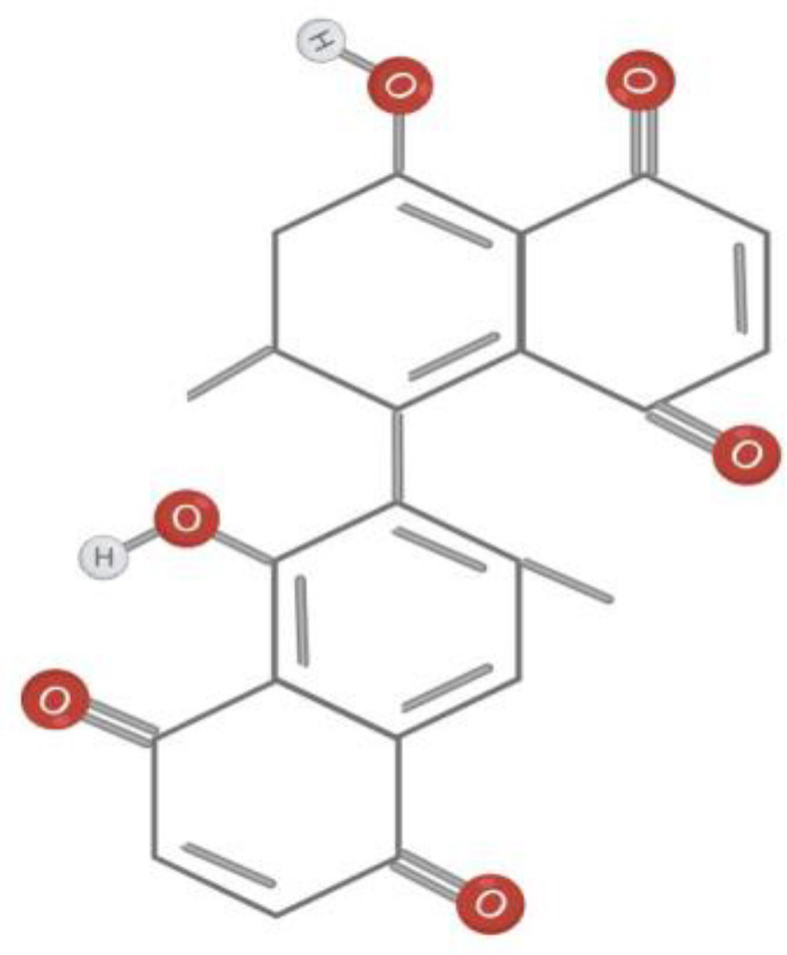
	Pentacyclic triterpene	2. β‐Amyrin	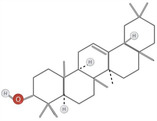
		3. Olean‐12‐en‐3‐one	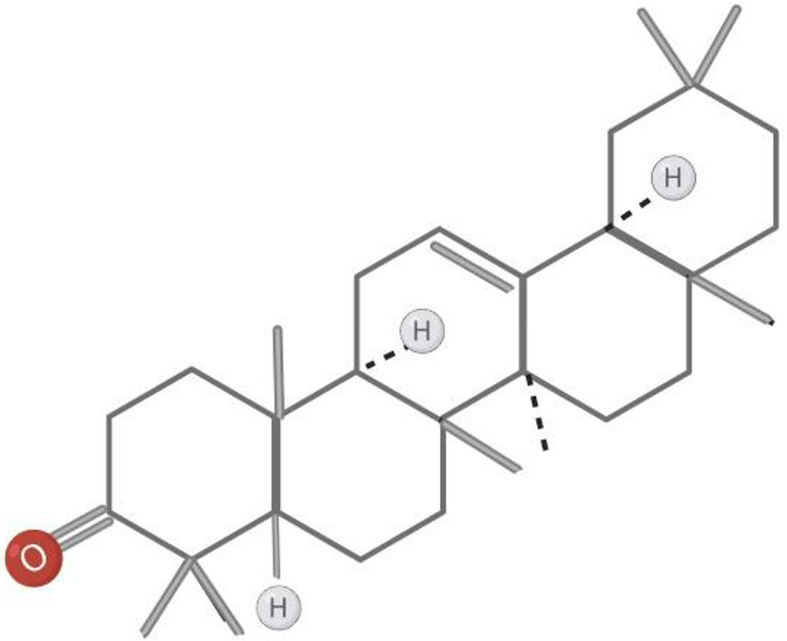
	Naphthoquinone	4. Bi‐naphthoquinone	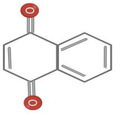
*D. tricolor*	Naphthoquinone epoxide	1. Diosquinone	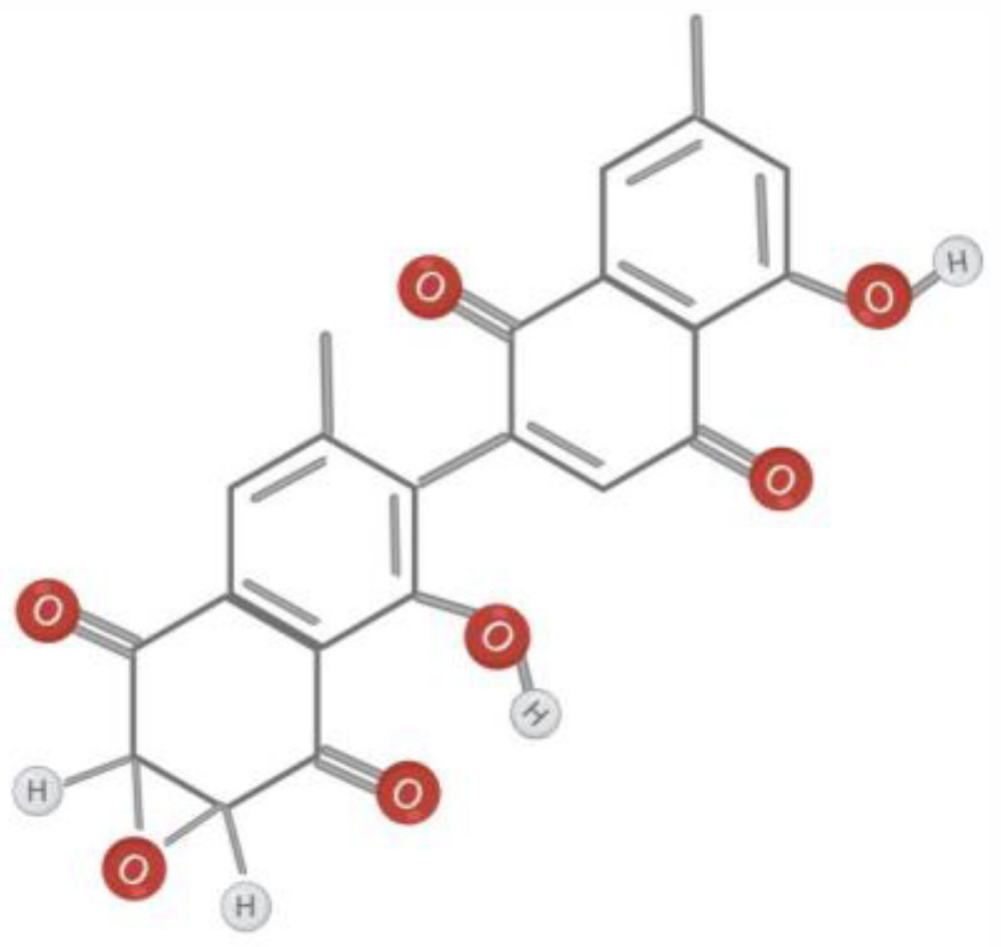
*D. usambarensis*	Naphthoquinones	1. Methyljuglone	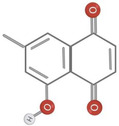
		2. Mamegakinone	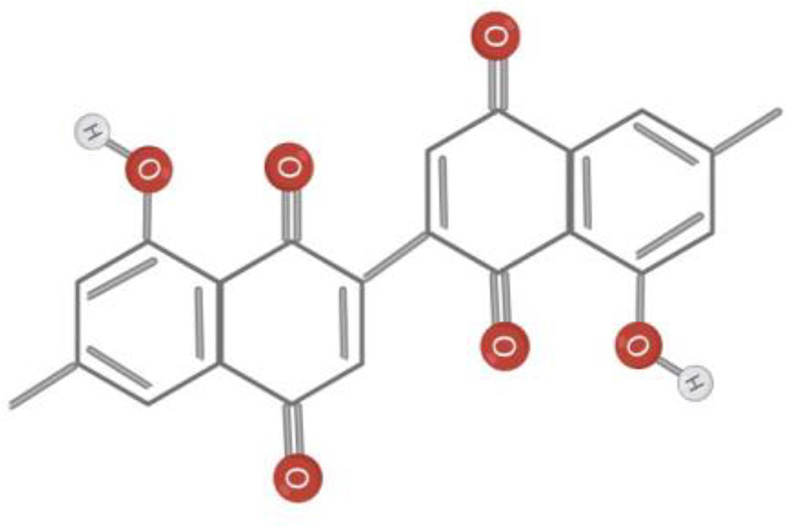
		3. Isodiospyrin	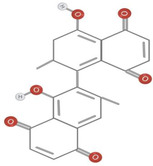

The presence of terpenoids, tannins, saponins, and flavonoids in *D. malabarica* was confirmed by qualitative phytochemical analysis employing a variety of solvents. In the phytochemical analysis, the blue–black color indicated the presence of tannins, while the frothy and yellow color indicated the occurrence of flavonoids and saponins, respectively. The eventual development of a reddish‐brown hue on the inner aspect of the leaf stated that the plant contained terpenoids. This phytochemical analysis confirmed that the various *D. malabarica* plant parts examined had a high concentration of flavonoids, crucial to the plant's antioxidant capacity (Alao et al., 2022). Similar phytochemicals, including terpenoids, saponins, and tannins, demonstrate that plants can inhibit the proliferation of bacteria and fungi. Likewise, saponins possess an anti‐obesity healing perspective (Sharifi‐Rad, 2016). Plants contain phytochemical substances that have been isolated and used to treat various health conditions and in the commercial production of dietary supplements and other nutrients. There were individual biological responses exhibited by each phytochemical, which could increase the likelihood of discovering new antibacterial components (Hassan & Ullah, 2019). Generally, phytochemicals positively affect human health, such as protection against disease, detoxification, infection, inflammation, and oxidation (Abdulhafiz et al., 2022). The significant in vitro antioxidant capacity of the ethanol extract of *D. malabarica* bark has been attributed to phytochemicals such as terpenoids and flavonoids. Priority research is being conducted on phytochemicals for dietary supplements and natural medications (Riaz et al., 2016). TPC and TFC values were also highest in methanol, suggesting that *D. malabarica* contains more polar phenolic compounds.

However, compared to other examined *D. malabarica* extracts, the bark extract contained the most significant concentrations of TFC and TPC. The phytochemical content (polyphenol, tannins, and flavonoids) is used to measure antioxidant capacity, and the results indicated that methanol bark extract, among other extracts, may be more effective in fighting free radicals (Chhetry et al., 2022; Othman et al., 2019). Polyphenols have been identified as the most potent antiviral, anti‐inflammatory, antioxidant, and antimicrobial agents among all phytochemicals (Ghosh et al., 2022). Antimicrobial and anti‐inflammatory properties and the ability to suppress tumor cell proliferation have been attributed to TFC and TPC. Previous research has also shown that phenolic chemicals play a significant role in *D. malabarica*'s high antioxidant activity (Ali et al., 2022). Flavonoids are essential polyphenols for antibiotic activity because only flavonoids form complexes with microbial proteins, cell walls, and numerous other biologically active components (Zreen et al., 2022).

## 4 ETHNOPHARMACOLOGICAL ASPECTS OF GENUS DIOSPYROS

Ethnopharmacology is the key to discovering new primitive medications from medicinal plants. Abd El Halim reports that the preponderance of *Diospyro*s species is found in tropical regions (Abd El Halim et al., 2014). All plant portions, including leaves, twigs, fruits, hardwood, barks, and roots, have been used to treat abdominal pain, asthma, dysentery, whooping cough, leprosy, menstrual problems, and dermatitis (Rauf et al., 2017).

Organs from various *Diospyros* species have been used in alternative medicine for decades. Ancient Chinese medical texts describe using persimmon leaves as a remedy, a nutritious drink, and a cosmetic ingredient, among other applications. There have been reports of the treatment of bleeding, burns, cardiovascular disease, chronic leg ulcers, internal bleeding, hematemesis, increased salivation, frostbite, lung distension, and snakebites (Xie et al., 2015; Zhang et al., 2017). The NaoXinQing tablet (a persimmon leaf extract tablet) has been registered as a trademark and included in the Chinese Pharmacopoeia for treating cerebral arteriosclerosis (Xie et al., 2015). In addition, the frosted leaves were described as an antihypertensive agent in traditional Japanese medicine, and the tea was widely consumed due to its antiaging properties, which are directly related to its high vitamin C content (Xie et al., 2015).

The bark of the well‐known species *D. discolor* was traditionally used to treat congestion, dysentery, diarrhea, and fever (Akter & Sarker, 2015). Other species, such as*D. peregrina*, were utilized to develop remedies for cholera, dysentery, diarrhea, diabetes, oral ulcers, and wounds (Saini et al., 2015). The unripe fruit juice of *Diospyros blancoi* is used as a natural remedy for diarrhea and as a first aid treatment for ulcers, and the bark, leaves, and roots are used to treat respiratory disorders and skin conditions, such as dermatitis (Khalipha et al., 2012; Morton, 1987). The stem and bark of *D. blancoi* were utilized as antioxidants, free radical scavengers, and anticancer agents (Khan et al., 2016). In Nigeria, it was reported that the roots of *D. mespiliformis* were used to treat malaria, and the bactericidal activity of its leaves and bark was observed. *Diospyros lycioides* has been used in Botswana for HIV/AIDS treatment (Rauf et al., 2017).

## 5 ANTICANCER POTENTIAL OF GENUS DIOSPYROS

Cancer has numerous causes and is exceedingly difficult to treat. Although toxicity to healthy cells should be avoided, it is essential for eliminating malignant cells. *Diospyros* species may prove beneficial in this context. Diospyrin and 8‐hydroxyisodiospyrin from *D. lotus* demonstrated remarkable anticancer activity. However, cancer is a heterogeneous, multifactorial complex disease, which the *Diospyros* compounds might not control (Rauf et al., 2017).

Han et al. reported that persimmon leaves inhibit nitrosamine‐induced squamous epithelial hyperplasia and malignancy in rats (Han et al., 1983). Park discovered an antitumor activity of persimmon leaves (Park et al., 1996). In A549 adenocarcinoma cells, persimmon leaf extract (PLE) and its galloylated homologs, such as PLEg (2"‐galloyl moiety), significantly increased the cytotoxicity of doxorubicin (DOX) by decreasing phosphorylation of the G2/M checkpoint (Kawakami et al., 2011). Different cancer cell lines were used to assess the anticancer potentials of an n‐hexane extract of *Diospyros maritima* Blume stems and were found to be very effective against all studied cell lines (Kuo et al., 1997). The diterpenoid diosmarioside D, isolated from methanol extracts of *D. maritima* leaves, exhibited potent cytotoxic activity against the lung adenocarcinoma (A549) cell line (Kawakami et al., 2018). Using an established NIH (National Institutes of Health) method, the cytotoxic potential of diosquinone, a naphthoquinone epoxide previously isolated from the bark of *D. mespiliformis* and *D. tricolor*, was assessed against 10 cancer cell lines. Diosquinone was found to be highly effective against a variety of cancer cell lines (Adeniyi et al., 2003). The IC50 (half‐maximal inhibitory concentration) values for the leaves, stem, and biosynthesized nanoparticles of *D. villosa* demonstrated their high cytotoxicity, revealing a potent inhibitory effect on the development of MCF‐7 and A549 cells (Table 2) (Adu et al., 2023).

**TABLE 2:** Anticancer potential of Genus Diospyros along with their proposed modes of action.

Species	Type of cancer	Cell lines	Part of plant	ED_50_	Mode of action	References
*D. lotus*	Human breast cancer	MCF‐7	Roots	2.5–20 μM	Exhibits oxidative stress‐dependent apoptosis; The increment in cytosolic calcium [Ca(2+)](c) which leads to the apoptotic cell death triggered by diospyrin diethylether	(Rauf et al., 2017)
*D. kaki*	Squamous epithelial hyperplasia	A549	Leaves	30 μg/mL	Reduces the phosphorylation of checkpoint proteins, such as structural maintenance of chromosome 1, checkpoint kinase 1, and p53 in DOX‐treated ataxia telangiectasia mutated in a dose‐dependent manner	(Han et al., 1983)
*D. maritima*	Hepatoma	HEPA‐3B	Stems	1.72 μg/mL	Cell growth inhibition	(Kawakami et al., 2018)
	Cervix carcinoma	HELA		1.92 μg/mL		
	Colon carcinoma	COLO‐205		2.24 μg/mL		
	Nasopharyngeal carcinoma	KB		1.85 μg/mL		
	Lung carcinoma	A549	Leaves	0.5 mg/mL		
*D. mespiliformis* and *D. tricolor*	Human breast cancer	BC‐1	Root bark	0.2 μg/mL	Inhibitory action against human cancer cell lines through induction of apoptosis and cell cycle arrest	(Adeniyi et al., 2003)
	Human fibrosarcoma	HT		0.2 μg/mL		
	Human lung cancer	LU‐1		0.2 μg/mL		
	Human colon cancer	COL‐2		3.1 μg/mL		
	Human nasopharyngeal carcinoma	KB		0.2 μg/mL		
	Viblastin non‐resistance human nasopharyngeal carcinoma	KB‐V(V + VLB)		1 μg/mL		
	Multiple drug resistance KB or viblastin‐resistant KB	KB‐V(V‐VLB)		1.7 μg/mL		
	Hormone‐dependent human prostrate cancer	LNCaP		4.5 μg/mL		
	Human glioblastoma	U373		0.18 μg/mL		
	Human neuroblastoma	SKNSH		0.2 μg/mL		
*D. villosa*	Human breast cancer	MCF‐7	Leaves	0.17 μg/mL	Induces apoptosis in cancer cells	(Adu et al., 2023)
			Stem	0.16 μg/mL		
			Nanoparticles	2.03 μg/mL		
	Human alveolar basal epithelial cancer	A549	Leaves	7.76 μg/mL		
			Stem	10.67 μg/mL		
			Nanoparticles	7.13 μg/mL		
	Human embryonic kidney	HEK293	Leaves	158.5 μg/mL		
			Stem	45.1 μg/mL		
			Nanoparticles	4.77 μg/mL		

### 5.1 In vitro studies of genus *Diospyros*

The organic plant extract of *Diospyros chamaethamnus* has a potent antiproliferative effect on cancer cell lines, according to in vitro testing. With respective IC50 values of 29.12, 16.08, and 24.67 μg/mL against UACC62 (melanoma cell line), TK10 (renal cell cancer), and MCF‐7 cells, *D. chamaethamnus* was the most potent (Dushimemaria et al., 2017). *Diospyros quercina* can be widely utilized in traditional Malagasy cancer treatment. *D. quercina* crude extract is an effective cytotoxic agent against P388 lymphocytic leukemia cell lines (Ruphin et al., 2014). The antiproliferative activity of *D. lotus* extract and isolated compounds against the inhibition of cell proliferation in nine human cancer cell lines, namely A375, ACHN (renal cell adenocarcinoma cell line), A549, CaCo‐2 (human colorectal adenocarcinoma cells), COR‐L23 (lung large cell carcinoma), MCF‐7, Huh‐7D12 (human hepatoma cell lines), and LNCaP (Lymph Node Carcinoma of the Prostate), was compared to the antiproliferative activity of one regular cell line, 142BR (Table 3). There was a dose–response relationship for each of the examined samples. With an IC50 of 12.20 μg/mL, *D. lotus* extract displayed the most significant inhibitory efficacy against COR‐L23 (Loizzo et al., 2009).

**TABLE 3:** Summary of various in vitro, in vivo, and in silico studies related to Genus Diospyros.

Species	Cell line	IC_50_ (μg/mL)	Potentials	References
In vitro studies				
*D. chamaethamnus* (organic extract)	UACC62	29.12	Antiproliferative effect	(Dushimemaria et al., 2017)
	TK10	16.08		
	MCF‐7	24.67		
*D. quercina* (crude extract)	P388	0.851 ± 0.050	Cytotoxic effect	(Ruphin et al., 2014)
*D. lotus* (methanolic extract)	A375	61.1 ± 2.5	Antiproliferative effect	(Loizzo et al., 2009)
	ACHN	>100		
	A549	48.6 ± 2.2		
	CaCo‐2	47.1 ± 2.8		
	COR‐L23	12.2 ± 1.1		
	MCF‐7	>100		
	LNCaP	>100		
	142BR	>100		
*D. fleuryana* (ethanolic extract)	KB	15.8	Cytotoxic effect	(Alex et al., 2012)
	Hep	29.75		
	Lu	53.33		
	MCF‐7	60.23		
*D. peregrina* (methanolic extract)	MCF‐7	53.32	Cytotoxic effect	(Ha et al., 2020)
	Hep G2	79.95		
*D. kaki* (crude extract)	HCT116	27.22	Cytotoxic effect	(Chen et al., 2020)
	A549	56.85		
	Hep G2	12.78		
	BT20	26.66		
	U2OS	48.72		
	MDB‐MA‐321	19.33		
*D. montana* (methanolic extract)	Hep G2	22	Anticancer effect	(Sujatha et al., 2023)
In vivo studies				
*D. kaki*	Human hepatocellular carcinoma	Flavonoids	Inhibits their proliferation via the PDGFR–Rac–JNK pathway	(Kim et al., 2020)
*D. kaki*	Mouse skin carcinogenesis	Naphthoquinone derivatives	Inhibitory effect on DMBA–TPA	(Kapadia et al., 1997)
*D. kaki*	Human lung non‐small carcinoma cells	Kaempferol	Apoptosis of NCI‐H460	(Leung et al., 2007)
*D. blancoi*	Mice Ehrlich ascites carcinoma cells	Saponins, triterpenes, tannins	Inhibition of EAC cells‐induced tumor‐bearing mice	(Howlader et al., 2012)

Species	Phytochemical	Docking score kcal/mol	Target protein	Protein residues	
In silico studies					
*D. kaki*	Acarbose	−7.4615	6CCY	GLU234, GLU278, and ASP292, ASN79; PHE161 and HIS194 LYS276 and LYS189	(Muhammad et al., 2022)
*D. kaki*	Quercetin 3‐O‐glucoside	−6.86404133	6CCY	GLU234; ASP291. LYS158 and GLU278	(Muhammad et al., 2022)

Rauf and colleagues discovered that the traditional medicinal plant *D. lotus* possesses significant cytotoxic activity and can be an effective anticancer medication (Rauf et al., 2021). The phytochemical analysis of the ethyl acetate (EtOAc) extract of *Diospyros fleuryana* leaves resulted in the isolation of 8′‐hydroxyisodiospyrin, a compound with anticancer potential (Alex et al., 2012). In both the Hep G2 (hepatocellular carcinoma) and MCF‐7 cell lines, the dichloromethane fraction of *D. peregrina* fruit extract exhibited the highest cytotoxicity (Ha et al., 2020). *D. kaki* inhibited the proliferation of human breast, colorectal, and hepatic cancer cells in vitro by inducing apoptosis and oxidative stress (Chen et al., 2020). Increased DNA damage, autophagy, and decreased mitochondrial membrane potential all contribute to the potent anticancer effect of silver oxide nanoparticles (Ag_2_ONPs) synthesized from the methanolic bark extract of *Diospyros montana* against hepatocellular carcinoma (Hep G2) cells (Sujatha et al., 2023).

### 5.2 In vivo studies of genus *Diospyros*

Hazara and coworkers examined the anticancer effects of diospyrin and its derivatives on 13 cancer cell lines. It was discovered that the acetylamine derivative increased cytotoxicity in HT‐29 colon cancer cells in particular. The findings suggest that cell death may involve mitochondrial pathways (Akter & Sarker, 2015). Park and colleagues' in vivo experiments on Sarcoma‐180 cells revealed the anticancer activity of *D. kaki* leaves (Park et al., 1996). *D. kaki* induces the apoptosis of cancer cells and inhibits their proliferation via the PDGFR–Rac–JNK (platelet‐derived growth factor receptor–Rac–c‐Jun N‐terminal kinase) pathway (Table 3) (Kim et al., 2020). In a two‐stage in vivo mouse skin carcinogenesis experiment, naphthoquinone derivatives, such as naphthazarin and juglone, exhibited potent inhibitory effects on promoting DMBA–TPA (7,12‐dimethylbenz(a)anthracene–TPA) tumors (Kapadia et al., 1997). The antioxidant system is activated by kaempferol, a pharmacologically active component of *D. kaki*, leading to apoptosis in human lung non‐small carcinoma cells (Leung et al., 2007; Nuzzo et al., 2022). Howlader and associates discovered cytotoxic effects in a *D. blancoi* ethanol extract in 2012 (Howlader et al., 2012). Using Ehrlich ascites carcinoma cells, Khan and coworkers evaluated the in vivo anticancer activity of *D. blancoi* and found that *D. blancoi* extracts showed substantial cytotoxic activity (Khan et al., 2016).

### 5.3 In silico studies of genus *Diospyros*

*In silico* docking is a useful method in computational drug discovery. It involves modeling the interactions between smaller molecules (drugs) and target protein receptors or enzymes to predict their binding affinity (Raza, Ahmad, et al., 2017; Raza, Jiang, et al., 2017; Raza, Khan, et al., 2017; Raza, Wei, et al., 2017). The extent of these interactions can affect the biosafety, pharmacological response, delivery rate, therapeutic efficiency, and the design of new drugs (Raza, Ahmad, et al., 2017; Raza, Jiang, et al., 2017; Raza, Khan, et al., 2017; Raza, Wei, et al., 2017). In silico investigations on the target enzymes validated the results of in vitro experiments, studies of binding orientation, and ligand–enzyme interactions. Muhammad and colleagues tested the effectiveness of *D. kaki* polyphenols in inhibiting AKT1 (AKT Serine/Threonine Kinase 1) (6CCY)‐driven cancer growth. The bioactive chemicals from *D. kaki* were found to inhibit AKT1 in an in silico analysis, suggesting they may have anticancer potential (Muhammad et al., 2022). Muhammad and colleagues examined the efficacy of *D. kaki* polyphenols in combating the cancer‐causing protein AKT1 (6CCY). In an *in silico* study, bioactive compounds derived from *D. kaki* were shown to inhibit AKT1, suggesting they have anticancer potential (Rauf et al., 2016). Novel anticancer dimeric naphthoquinones derived from *D. lotus* exhibit significant anticarcinogenic activity due to their superior docking statistics compared to the norm (Rauf et al., 2021).

## 6 CLINICAL STUDIES

Human body extensively uses ligands such as natural compounds from plants which have brilliant affinities toward transporter proteins like human serum albumin, lipoprotein, and glycoprotein (Raza, Ahmad, et al., 2017; Raza, Jiang, et al., 2017; Raza, Khan, et al., 2017; Raza, Wei, et al., 2017). Drug resistance has become a critical global issue. Consequently, the discovery of latest, less lethal drugs provides a basic and urgent implication in the field of drug discovery (Raza, Ahmad, et al., 2017; Raza, Jiang, et al., 2017; Raza, Khan, et al., 2017; Raza, Wei, et al., 2017). In their study, Kaushik and colleagues found that by inhibiting the proliferation of regulatory T (Treg) cells in the microenvironment of breast and lung cancer tumors, *D. peregrina* significantly boosts the immune system's ability to protect against these diseases (Kaushik et al., 2022). Roy et al. demonstrated that *D. peregrina*‐mediated immunomodulation of lymphocytes isolated from the blood of breast cancer patients promotes lymphocytic proliferation, induces type 1 cytokines (IL‐12 (interleukin‐12), IFN‐γ (interferon gamma)), and produces the tumor‐killing agent nitric oxide (NO) (Roy et al., 2021). Harun Al Rashid and his colleagues investigated the anticancer properties of *Diospyros melanoxylon* Roxb. They discovered that ursolic acid isolated from *D. melanoxylon* inhibits cancer development, progression, and metastasis in multiple cancer types, making it an effective cancer prevention and treatment agent (Al Rashid et al., 2017). *D. malabarica* fruit preparation (DFP) upregulates the expression of type 1 specific cytokines and enhances tumor suppression by modulating several epigenetic markers to elicit a protective immune response against tumors (Bhootra et al., 2023).

## 7 MOLECULAR MECHANISM

Gewirtz hypothesized in 1999 that the primary lethal mechanism of the quinonoid class of synthetic substances is bioreduction, followed by interaction with molecular oxygen and the formation of reactive oxygen species (ROS) (Gewirtz, 1999). Kayashima and colleagues first proposed apoptosis as a form of cell death caused by reactive oxygen species (ROS) in 2009 (Kayashima et al., 2009). It is becoming increasingly apparent that reactive oxygen species (ROS) play a crucial role in mediating the apoptotic process, which quinonoid compounds employ as an anticancer strategy (Lee et al., 2012). Kumar et al. demonstrated that the bisnaphthoquinonoid derivative diospyrin diethylether induces apoptosis in response to oxidative stress in various human cancer cell lines and tumor models. It was discovered that diospyrin diethylether induces apoptosis in MCF‐7 human breast cancer cells; consequently, the researchers examined the effect of an increase in cytosolic calcium [Ca(2+)] (c) on this process. In addition, they have cast light on the redox signaling induced by diospyrin diethylether, which integrates calcium‐dependent calpain/caspase 12 activation and mitochondrial changes to highlight the induction of apoptotic cell death (Kumar et al., 2012). According to studies, plumbagin inhibits angiogenesis and tumor formation in human umbilical vein endothelial cells (HUVECs) in vitro and mouse models of human colon carcinoma and prostate cancer. Following vascular endothelial growth factor receptor‐2 (VEGFR2) activation, the rat sacroma (Ras) signaling pathway was the intervening mechanism (Lai et al., 2012). According to Nguyen and colleagues’ findings, kaempferol could induce apoptosis in the lung cancer cell line (A459) and inhibit the mitogen‐activated protein kinase (MAPK) phosphorylation pathway and c‐Fos and nuclear factor of activated T cells (NFATc) in bone marrow cells (Nguyen et al., 2003). In U‐2OS human osteosarcoma cells, kaempferol could significantly inhibit the mRNA expression levels of extracellular signal‐regulated kinase (ERK), JNK, and p38 proteins. Phosphatidylinositol‐3‐kinase (PI3K) direct binding efficacy of kaempferol in inhibiting PI3K/Akt (protein kinase B) pathway, thereby inhibiting nuclear factor kappa B (NF‐κB) and activator protein 1 (AP‐1) activities, has been confirmed, suggesting its role in cellular functions, including angiogenesis and apoptosis (Choudhary et al., 2023).

## 8 CONCLUSION

The *Diospyros* genus is considered a promising source of potent anticancer constituents comprising diverse phytochemicals with inherent therapeutic properties. In this review, the detailed investigation underlines the multidimensional nature of this genus, covering not only its rich phytochemistry but also highlighting its significance against various cancers. The comprehensive evidence derived from multiple in vitro, in vivo, and in silico studies explained in this article reinforces the fact that *Dios*pyros species possess several bioactive compounds, which can be further explored in different clinical trials to validate their use as cancer therapeutics. As the scientific community strives to bridge the gap between traditional knowledge and modern research, the Genus Diospyros may provide a new paradigm in nature's pharmacopoeia, justifying sustained exploration and scrutiny for its invaluable contributions to anticancer drug discovery.

## AUTHOR CONTRIBUTIONS

**Abdur Rauf:** Conceptualization (lead); data curation (lead); investigation (lead); methodology (lead); validation (equal); visualization (equal); writing – original draft (lead); writing – review and editing (equal). **Zuneera Akram:** Conceptualization (equal); investigation (supporting); methodology (supporting); validation (supporting); visualization (equal); writing – review and editing (supporting). **Nabia Hafeez:** Investigation (supporting); methodology (supporting); validation (equal); visualization (equal); writing – review and editing (supporting). **Anees Ahmed Khalil:** Conceptualization (supporting); data curation (supporting); investigation (equal); methodology (equal); validation (equal); visualization (supporting); writing – original draft (equal); writing – review and editing (equal). **Ahood Khalid:** Investigation (supporting); methodology (supporting); validation (supporting); visualization (equal); writing – review and editing (supporting). **Zoya Abid:** Methodology (supporting); validation (supporting); visualization (supporting); writing – review and editing (supporting). **Hassan A. Hemeg:** Data curation (supporting); investigation (supporting); methodology (supporting); validation (equal); visualization (equal); writing – review and editing (supporting). **Abdullah S. M. Aljohani:** Investigation (supporting); methodology (supporting); validation (equal); visualization (equal); writing – review and editing (supporting). **Waleed Al Abdulmonem:** Investigation (supporting); methodology (supporting); validation (equal); visualization (equal); writing – review and editing (supporting). **Mohammed Mansour Quradha:** Conceptualization (equal); investigation (supporting); methodology (supporting); validation (equal); visualization (equal); writing – original draft (supporting); writing – review and editing (supporting).

## CONFLICT OF INTEREST STATEMENT

The authors declare no conflict of interest.

## Data Availability

The dataset supporting the conclusions of this article is included within the report.
